# Serial Measurements of N-Terminal Pro-Brain Natriuretic Peptide in Patients with Coronary Heart Disease

**DOI:** 10.1371/journal.pone.0117143

**Published:** 2015-01-28

**Authors:** Dhayana Dallmeier, Michael J. Pencina, Iris Rajman, Wolfgang Koenig, Dietrich Rothenbacher, Hermann Brenner

**Affiliations:** 1 University of Ulm Medical Center, Department of Internal Medicine II-Cardiology, Ulm, Germany; 2 Department of Epidemiology, Boston University School of Public Health, Boston, MA, United States of America; 3 Ulm University, Institute of Epidemiology and Medical Biometry, Ulm, Germany; 4 Duke Clinical Research Institute, Department of Biostatistics and Bioinformatics, Duke University, Durham, NC, United States of America; 5 Novartis Institute of Biomedical Research, Basel, Switzerland; 6 German Cancer Research Center, Division of Clinical Epidemiology & Aging Research, Heidelberg, Germany; Medical University Hamburg, University Heart Center, GERMANY

## Abstract

**Objective:**

To assess the prognostic value of 12-months N-Terminal Pro-Brain Natriuretic Peptide (NT-proBNP) levels on adverse cardiovascular events in patients with stable coronary heart disease.

**Methods:**

NT-proBNP concentrations were measured at baseline and at 12-months follow-up in participants of cardiac rehabilitation (median follow-up 8.96 years). Cox-proportional hazards models evaluated the prognostic value of log-transformed NT-proBNP levels, and of 12-months NT-proBNP relative changes on adverse cardiovascular events adjusting for established risk factors measured at baseline.

**Results:**

Among 798 participants (84.7% men, mean age 59 years) there were 114 adverse cardiovascular events. 12-months NT-proBNP levels were higher than baseline levels in 60 patients (7.5%) and numerically more strongly associated with the outcome in multivariable analysis (HR 1.65 [95% CI 1.33–2.05] vs. HR 1.41 [95% CI 1.12–1.78], with a net reclassification improvement (NRI) of 0.098 [95% CI 0.002–0.194] compared to NRI of 0.047 [95% CI −0.0004–0.133] for baseline NT-proBNP levels. A 12-month 10% increment of NT-proBNP was associated with a HR of 1.35 [95% CI 1.12–1.63] for the onset of an adverse cardiovascular event. Subjects with a 12-month increment of NT-proBNP had a HR of 2.56 [95% CI 1.10–5.95] compared to those with the highest 12-months reduction.

**Conclusions:**

Twelve-months NT-proBNP levels after an acute cardiovascular event are strongly associated with a subsequent event and may provide numerically better reclassification of patients at risk for an adverse cardiovascular event compared to NT-proBNP baseline levels after adjustment for established risk factors.

## Introduction

Cardiovascular diseases (CVD) are the number one cause of death worldwide. About 17.5 million people died from CVD in 2008, 7.3 million due to coronary heart disease (CHD) and 6.2 million due to stroke[1]. About 19% of men and 26% of women subjects older than 45 years having a myocardial infarction (MI) will die within one year, whereby survivors will have a 1.5 to 15 times higher risk for illness and death compared to the general population[2]. In such a scenario the evaluation of biomarkers that could improve risk stratification and clinical decision making among patients with prevalent CHD becomes essential.

N-Terminal Pro-Brain Natriuretic Peptide (NT-proBNP) is a marker of myocardial hemodynamic stress. It is released from the myocardium in the settings of volume and/or pressure overload, improving myocardial relaxation, counteracting vasoconstriction, sodium retention and antidiuretic effects of the activated renin-angiotensin-aldosteron system[3]. Single measurements of NT-proBNP have shown strong prognostic value for cardiovascular events (CVE), all-cause and CVD-specific mortality or heart failure (HF) in cardiac patients[4,5,6] as well as in the general population[7,8], providing modest improvements in risk discrimination after adjustment for conventional cardiovascular (CV) risk factors[9]. Longitudinal changes of NT-proBNP have been examined mostly for intervals shorter than 12 months[10,11]. So far, a decrease in NT-proBNP concentrations >30% has been perceived as evidence of therapeutic effectiveness in patients with chronic HF[12,13]. In community-dwelling elderly people NT-proBNP changes over two to three years were associated with changes in patient risk for a CVE independent of CV risk factors, ejection fraction, or medication use[14]. Serial determinations of natriuretic peptides have been proposed for prognostic assessment in CV patients[15], but still there is limited information about the within-person variability in natriuretic peptide levels over time[9], and the prognostic value of NT-proBNP among patients with stable CHD[5,16], whose frequency of follow-up examinations depends mainly on the likelihood of disease progression and the occurrence of a subsequent CVE.

The present study evaluates the prognostic value of 12-months levels of NT-proBNP and the estimated 12-month longitudinal change in NT-proBNP with respect to the onset of adverse CVE during nine years follow-up in patients with stable CHD.

## Methods

### Study Population

The KAROLA cohort is a prospective study including 1204 patients with CHD aged 30–70 years who were admitted within three months after an acute coronary syndrome (ACS) or coronary artery bypass graft (CABG) to a three-week in-hospital cardiac rehabilitation program at two co-operating hospitals in Southwest Germany between January 1999 and May 2000. For this study following exclusion criteria applied: occurrence of an adverse CVE (n = 61) or censored (n = 149) prior to 12-months follow-up, number of affected vessels recorded as 0 (n = 12), missing values for NT-proBNP at baseline and / or at 12-months follow-up (n = 65); for left ventricular function at baseline (n = 97), for lipid profile (n = 15), for creatinine (n = 4) for medications (n = 2) and for blood pressure (n = 1). Thus, a total of 798 participants remained for this analysis.

The study was approved by the ethical committees of the Universities of Ulm and Heidelberg and of the Physicians’ Chambers of the States of Baden-Württemberg and Hessen and has been performed in accordance with the ethical standards laid down in the 1964 Declaration of Helsinki and its later ammendments. All participants gave written informed consent.

### Data collection

At the beginning of the rehabilitation program, all participants filled out a standardized questionnaire containing socio-demographic information, medical history and assessment of lifestyle factors, and were weighed, measured and examined by a physician. Body mass index (BMI) was defined as the individual’s body weight divided by height squared.

Baseline fasting blood samples were obtained at the end of the rehabilitation. Serum samples underwent centrifugation within two hours, and were aliquoted in four aliquots a two ml. Samples were stored for up to one month at −20°C, and then transferred to a −80°C freezer until analysis. Serum from 12-months follow-up was collected by the primary care physicians (PCP) and sent after centrifugation to the study centre. Obtained aliquots were stored at −80°C until analysis. Plasma NT-proBNP was measured using a one-step enzyme immunoassay based on electrochemiluminescence (Roche Elecsys 2010; Roche Diagnostics, Mannheim, Germany, inter-assay coefficient of variation (CV) 3–7%). Baseline levels of NT-proBNP were analyzed in 2003, 12-months follow-up in 2011. We evaluated the eight year stability of NT-proBNP in serum samples which had been stored at −80°C and experienced maximum one thaw cycle, with an estimated recovery within the range of 89.5% to 103.0%. Serum creatinine was measured by the kinetic Jaffe method in one hospital (Center 1, n = 395, inter-assay CV 2.4–5.7%), whereas in the other hospital an enzymatic p-aminophenazone method was used (Center 2, n = 403, inter-assay CV 1.2–2.2%). All marker measurements were performed in a blinded fashion.

Left ventricular (LV) function at baseline was assessed using either the most recent LV angiography (n = 707) or available echocardiographic examinations (n = 644). When both examinations were available the one reporting the lower ejection fraction (EF) was considered, and the LV function was categorized into normal (>65%), mild (50–65%), moderate (35–50%) or severe depression (<35%). Prescribed medications at discharge were recorded.

### Adverse Cardiovascular Disease Events

**Fig 1:**
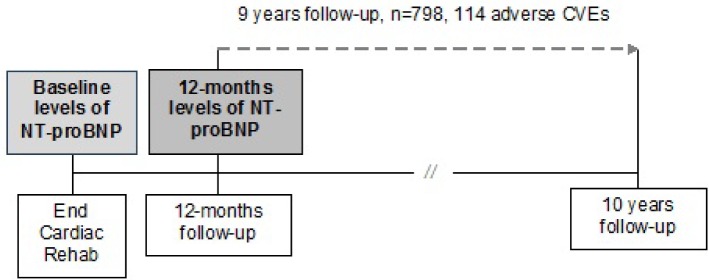
Study design.

Active follow-up was conducted 1, 3, 4.5, 6, 8, and 10 years after discharge from the rehabilitation centers using mailed standardized questionnaires. Primary care physicians reported on the same time intervals any adverse CVE (non-fatal MI and/or stroke) and new treatment and/or diagnoses. If a patient deceased, the main cause of death was extracted from death certificates obtained from the local Public Health Department and coded according to the International Classification of Diseases (ICD-9 pos. 390–459 until 2003, followed by ICD-10 pos. I0-I99 or R57.0). For the purpose of this analysis follow-up started at the corresponding date for 12-months follow-up questionnaire for a maximal length of nine years (**Fig. 1**).

### Statistical Analysis

Discrete variables are presented as numbers and percentages, continuous variables as arithmetic means and standard deviations or medians and/or inter-quartile range (IQR) based on their distribution. The median follow-up estimate was based on the reverse Kaplan-Meier estimator[17]. NT-proBNP concentration showed a skewed distribution at baseline and at 12-months follow-up. We calculated the median of NT-proBNP for the various covariates levels, and tested the respective associations with NT-proBNP using nonparametric methods (Wilcoxon rank sum test, Kruskal-Wallis test). For further analysis, NT-proBNP levels were natural log-transformed (ln). We created a categorical variable for blood pressure as defined in the Framingham Risk Score chart for 10-year risk for CHD[18]. We estimated the glomerular filtration rate (eGFR) according to the Chronic Kidney Disease Epidemiology (CKD-EPI) equation. Due to statistically significant differences for creatinine levels between both centers (center 1 mean 1.05, std 0.41, center 2 mean 0.83, std 0.2), eGFR levels were standardized (mean 0, SD 1) for each center and pooled together.

Using Cox-proportional hazards models we evaluated the prognostic value of ln NT-proBNP at baseline (**Model 1**) and at 12-months follow-up (**Model 2**) with adverse incident CVEs after adding them to a defined prognostic model adjusted for well-established CV risk factors measured at baseline: age, sex, BMI, blood pressure, LV function, smoking, history of diabetes, total cholesterol, HDL-Cholesterol, standardized eGFR, and use of statins (**basic baseline prognostic model**). We checked the assumption of proportional hazards by including time-dependent interactions of each variable with the natural logarithm of survival time. We evaluated improvement in model fit using the Likelihood Ratio. We assessed the predictive value’s improvement by estimating the increment in the C-statistic, the continuous Net Reclassification Improvement (NRI) as well as the NRI according to the risk strata of <10%, 10–20%, and >20% of predicted probability for an adverse CVE[19,20], and the Integrated Discrimination Improvement (IDI). We provide the estimated NRIs obtained after addition of 1) ln NT-proBNP at baseline (**Model 1**) and 2) ln NT-proBNP levels at 12-months follow-up (**Model 2**) to the **basic baseline prognostic model**.

We estimated the individual relative 12-months change of NT-proBNP defined as [(12-month ln NT-proBNP – baseline ln NT-proBNP) / baseline ln NT-proBNP] * 100. Using Cox-proportional hazards models we evaluated the association between these 12-months relative changes of ln NT-proBNP as a continuous variable (**Model 3**) and in categories (**Model 4**) with the onset of adverse CVE after adjustment for baseline levels of ln NT-proBNP, age and sex, and additional adjustment for BMI, blood pressure, LV function, smoking, history of diabetes, total cholesterol, HDL-Cholesterol, standardized eGFR, and use of statins. For categorical analysis we built an upper category including those with a 12-months relative increment of ln NT-proBNP, dividing the rest of the sample in tertiles. We evaluated the prognostic utility of the 12-months relative change by assessing the improvements obtained in the C-statistic, NRI and IDI in **Model 3** and **Model 4**.

We assessed the internal validity of the obtained estimates for NRI and IDI by performing a 10-fold cross-validation. The degree of over-optimism was not substantial and for any metric did not reduce the metric by a relative amount of at most 7.5%. We considered p<0.05 as statistically significant. All analyses were performed using SAS software, version 9.2 (SAS Institute Inc., Cary, North Carolina).

## Results

**Table 1** shows the baseline clinical characteristics of the 798 study participants. The mean age was 59 ± 8 years, 84.7% of the study sample were men; 55.5% were overweight, and 17% obese. Only 3.9% reported current smoking at baseline. Diabetes was present among 16.2% of participants. Moderate and severe depression of LV function was documented for 15.3% and 5.8% respectively. The median time from primary event to blood withdrawal was 43 days (IQR 36 to 52 days). Between sexes the median levels for NT-proBNP were higher among females at baseline (690.2 versus 542.5 pg/mL) and at one-year follow-up (270.0 versus 179.5 pg/mL). Log-transformed NT-proBNP at baseline and one-year follow-up showed an age and sex adjusted Pearson correlation coefficient equal to 0.735 (p-value <0.001).

**Table 1:** Sociodemographic, clinical and laboratory characteristics of participants (n = 798).

Age (year) mean ± std	59 ± 8
**Male** n (%)	676 (84.7)
**Body Mass Index** (kg/m^2^) mean ± std	27.0 ± 3.5
**Body Mass Index** n (%)	
Normal weight	221 (27.7)
Overweight	443 (55.5)
Obese	134 (17)
**Smoking** n (%)	
Never smoker	262 (32.8)
Ex-smoker	505 (63.3)
Current smoker	31 (3.9)
**History of Myocardial Infarction** n (%)	405 (50.8)
**Coronary Artery Bypass Graft** n (%)	395 (49.5)
**History of Diabetes** n (%)	129 (16.2)
**Left Ventricular Function** n (%)	
Normal	434 (54.4)
Mild depression	196 (24.6)
Moderate depression	122 (15.3)
Severe depression	46 (5.8)
**Systolic Blood Pressure** (mmHg) mean ± std	120 ± 15
**Diastolic Blood Pressure** (mmHg) mean ± std	73 ± 9
**Blood Pressure** ^**a**^ n (%)	
0 points	522 (65.4)
1 point	133 (16.7)
2 points	129 (16.2)
3 points	14 (1.8)
**Time to blood withdrawal** median, (Q1, Q3)	43 (36, 52)
**Total Cholesterol** (mg/dl) mean ± std	168 ± 32
**HDL Cholesterol** (mg/dl) mean ± std	40 ± 11
**NT-proBNP at baseline** (pg/mL) median, (Q1, Q3)	
Female	690.2 (357.7, 1644)
Male	542.5 (277.6, 1043.5)
**NT-proBNP at one year follow-up** (pg/mL) median, (Q1, Q3)	
Female	270 (115, 491)
Male	179.5 (87.4, 373.5)

^a^ according to FRS chart for 10 years risk for CHD.

Compared to those excluded due to a follow-up time shorter than 12 months (n = 210) the study sample was older, had a higher proportion of women, never smokers, and normal LV function, with a lower prevalence of diabetes and lower levels of NT-proBNP (median) and total cholesterol (mean) at baseline (**S1 Table**).

NT-proBNP levels at baseline and at 12-months follow-up were noted to be different across the strata for the following variables: sex, age, BMI, standardized eGFR, LV function, and use of statins. Only 12-months NT-proBNP levels vary across the strata of diabetes and blood pressure. We did not detect any differences in the median levels of NT-proBNP at baseline with respect to the time since the acute event to blood withdrawal **(S2 Table)**.

During follow-up there were a total of 114 adverse CVD events (median follow-up 8.96 years, [95% CI 8.95, 8.97]). The crude incidence was 19.5 per 1000 patient-years [95% CI 16.1, 23.3]. The nine-year observed probability of an event was equal to 0.22. Addition of ln NT-proBNP measured at 12-months follow-up to our **basic baseline prognostic model** (**Model 2**) was associated with a numerically higher hazard for an adverse CVE compared to **Model 1** which instead included baseline levels of ln NT-proBNP (HR per one unit increase in ln NT-proBNP representing 2.72-fold increase in NT-proBNP: 1.65 [95% CI 1.33, 2.05] in **Model 2** versus HR 1.41 [95% CI 1.12, 1.78] in **Model 1**) (**Table 2**). When including both ln baseline and 12-months levels of NT-pro BNP in the **basic baseline prognostic model**, a one unit increment of ln 12-months levels was associated with a HR 1.64 [95% CI 1.24, 2.17], while the baseline levels were no more associated with the outcome (HR 1.01 [95% CI 0.75, 1.36]).

**Table 2:** Cox-proportional hazards models (n = 798, 114 adverse cardiovascular events).

	Models adjusted for age and sex		Fully adjusted model^a^	
	HR [95% CI]	*P*-value	HR [95% CI]	*P*-value
Baseline levels of ln NT-proBNP (**Model 1**)	1.73 [1.44, 2.06]	<0.001*	1.41 [1.12, 1.78]	0.003*
12-months levels of ln NT-proBNP (**Model 2**)	1.92 [1.61, 2.28]	<0.001*	1.65 [1.33, 2.05]	<0.001*

^a^ adjusted for age, sex, BMI, blood pressure, LV function, current smoking, diabetes, total cholesterol, HDL-cholesterol, standardized eGFR, and use of statins.

Our **basic baseline prognostic model** showed a C-statistic equal to 0.69 [95% CI 0.64, 0.74]. We observed a modest improvement in C-statistics to 0.71 [95% CI 0.66, 0.76] and 0.72 [95% CI 0.67, 0.77]) after addition of the baseline (**Model 1**) and 12-months levels of ln NT-proBNP (**Model 2**) respectively.

Similarly, the continuous NRI was numerically larger when 12-month ln NT-proBNP was added to the basic baseline prognostic model (**Model 2**) (0.597 versus 0.487). According to the analysis considering the risk strata (<10%, 10–20%, >20%) the addition of 12-months ln NT-proBNP levels resulted in a 0.009 net upwards reclassification among cases (event NRI), as well as in a 0.089 net downwards reclassification among non-cases (non-event NRI), for an NRI of 0.098 [95% CI 0.002, 0.194]. In contrast, when baseline ln NT-proBNP was added to the basic prognostic model the event, non-event and overall NRIs were 0.026, 0.020 and 0.047 [95% CI 0.039, 0.133] respectively (**Model 1**). With respect to improvement in the prediction slope we obtained the best IDIs in the models containing either 12-months level of ln NT-proBNP or a 12-months change of ln NT-proBNP (**Table 3**).

**Table 3:** Measures of model accuracy.

	Basic baseline prognostic model^a^	Basic baseline prognostic model plus baseline levels of ln NT-proBNP^a^ (Model 1)	Basic baseline prognostic model plus 12 months levels of ln NT-proBNP^a^ (Model 2)	Model 1 plus 12 months relative changes of ln NT-proBNP as continuous variable^b^ (Model 3)	Model 1 plus 12 months relative change of ln NT-proBNP in categories^b^ (Model 4)
Model fit					
Likelihood Ratio (χ2) (df)	78.2 (17)	87.02 (18)	98.9 (18)	97.4 (19)	96.4 (21)
Discrimination					
C-statistic [95% CI]	0.69 [0.64, 0.74]	0.71 [0.66, 0.76]	0.72 [0.67, 0.77]	0.72 [0.67, 0.77]	0.71 [0.66, 0.76]
Reclassification					
Integrated Discrimination Improvement [95% CI]		0.019 [0.006, 0.033]	0.047 [0.027, 0.066]	0.043 [0.025, 0.061]	0.039 [0.022, 0.056]
Continuous NRI		0.487	0.597	0.568	0.535
NRI [95% CI] according to risk strata <10%, 10–20%, > 20%		0.047 [−0.039, 0.133]	0.098 [0.002, 0.194]	0.086 [−0.009, 0.182]	0.077 [−0.015, 0.170]
Expected number of subjects to be reclassified					
Subjects with CVD-event	n_up_/n_down_	12/9	13/12	13/12	11/12
Subjects without CVD.event	n_up_/n_down_	65/79	80/141	83/136	77/136

^a^adjusted for age, sex, BMI, blood pressure, LV function, current smoking, history of diabetes, total cholesterol, HDL-cholesterol, GFR std, use of statins

^b^adjusted for baseline levels of ln NT-proBNP, age, sex, BMI, blood pressure, LV function, current smoking, history of diabetes, total cholesterol, HDL-cholesterol, GFR std, use of statins

**Fig 2:**
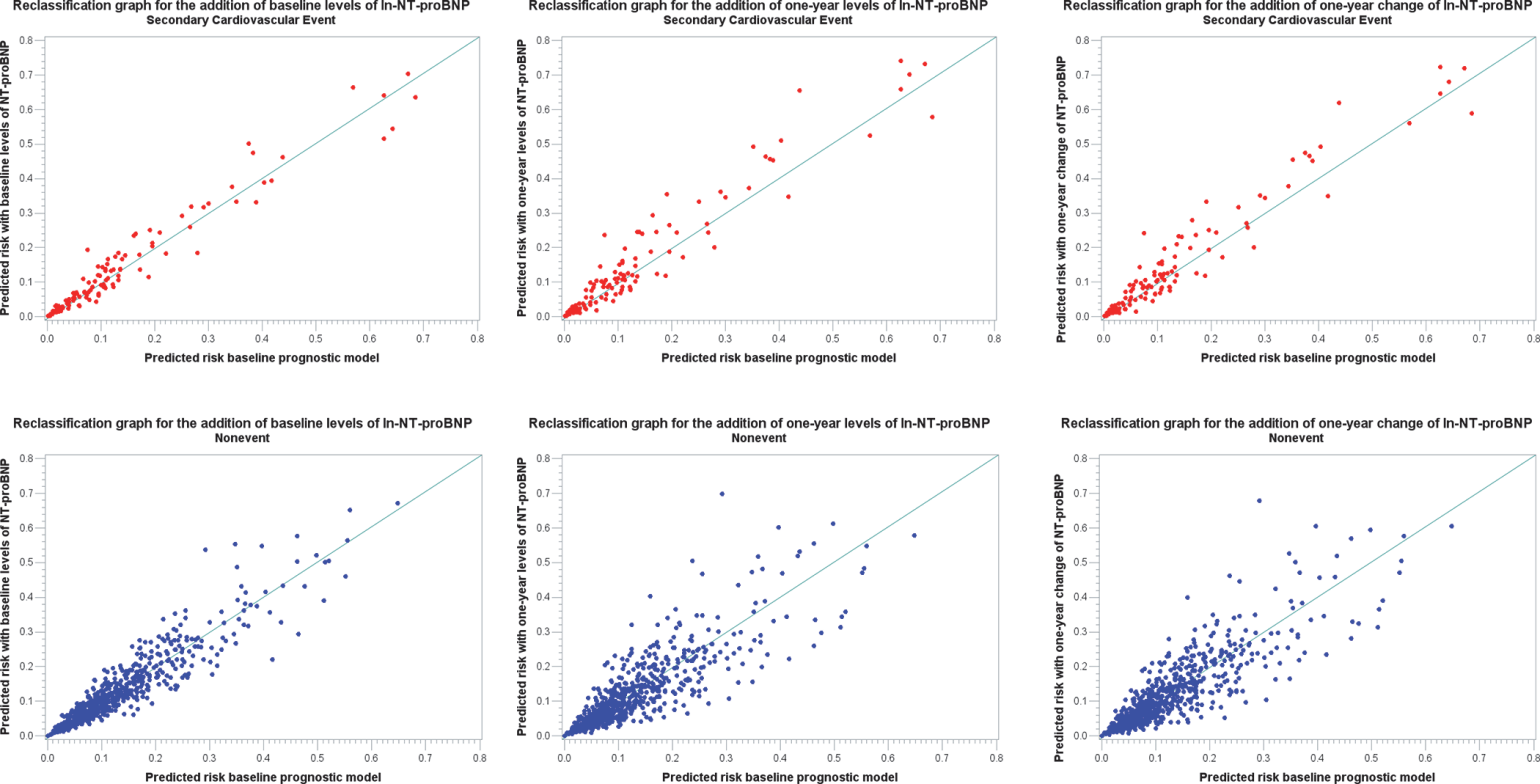
Reclassification graphs for a 20% risk cut-off in Models 1, 2 and 3.

**Fig. 2** is showing the reclassification graphs for a 20% risk cut-off after the addition of baseline levels of ln NT-proBNP (**Model 1**), 12-months levels of ln NT-proBNP (**Model 2**), and the 12-months change of ln NT-proBNP as a continuous variable (**Model 3**) to the baseline prognostic model.

### Relative Change of NT-proBNP

**Fig 3:**
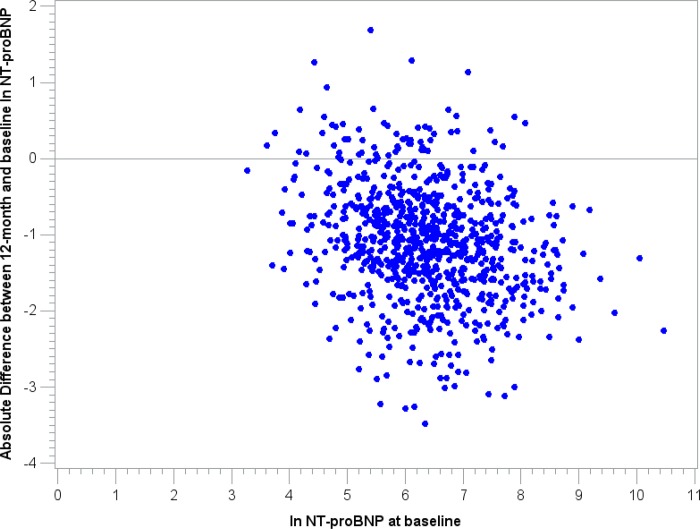
Scatter-plot for absolute differences between 12-month and baseline ln NT-proBNP.

**Fig 4:**
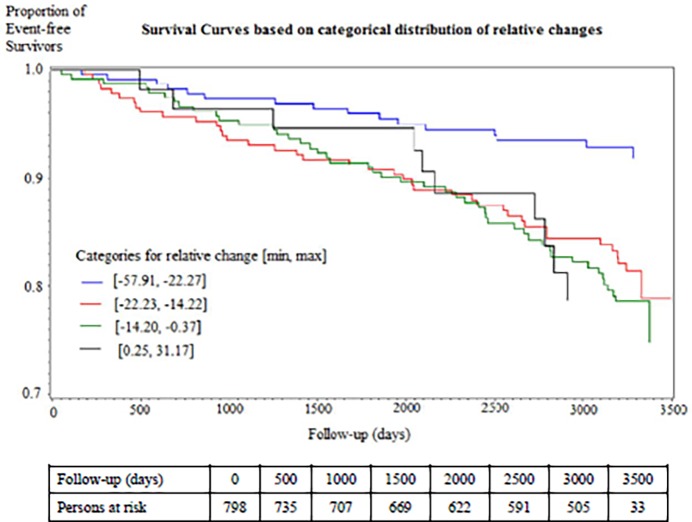
Kaplan-Meier estimates of adverse cardiovascular events according to categories of relative changes (*P*-value of log rank test = 0.001).

Overall, the median relative 12-months change of ln NT-proBNP was −17.2%. A total of 738 (92.5%) participants showed a decrease of NT-proBNP, while only 60 subjects showed a 12-month increment of NT-proBNP (**Fig. 3**). A 12-month 10% increment of ln NT-proBNP was associated with a HR of 1.35 [95% CI 1.12, 1.63] for the onset of an adverse CVE in the multivariable analysis including ln baseline levels of NT-proBNP (**Model 3**). Survival analysis showed a statistically significant difference among the categories of relative change (Log-Rank p-value = 0.001) (**Fig. 4**).We observed a lower incidence rate among those with the highest reduction [8.1 events per 1000 person-years], while those with a 12-months increment of ln NT-proBNP had an incidence rate of 23.4 events per 1000 person-years. In the multivariable analysis (**Model 4**) we observed a steady increase in the hazard when comparing the 2^nd^, 3^rd^, and 4^th^ category to the bottom one with the highest relative reduction over 12 months (HR: 2.02 [95% CI 1.11, 3.69], 2.26 [95% CI 1.25, 4.08], and 2.56 [95% CI 1.10, 5.95], p-value for Trend 0.008) (**Table 4**).

**Table 4:** Relative change of ln NT-proBNP by categories, Cox-proportional hazards models (n = 798, 114 adverse cardiovascular events).

Category [min, max]^a^	Events/ Subjects	Incidence Rate (per 1000 person-years)	Log-Rank Test	Adjusted for log-transformed baseline levels of NT-proBNP, age and sex.		Fully adjusted model (Model 4)^b^.	
				HR [95% CI]	*P*-value	HR [95% CI]	*P*-value
1 [−57.91, −22.27]	16/246	8.8	0.001	Reference			
2 [−22.23, −14.22]	41/246	23.0		2.01 [1.11, 3.61]	0.020*	2.02 [1.11, 3.69]	0.022*
3 [−14.20, −0.37]	47/246	25.6		2.37 [1.33, 4.23]	0.003*	2.26 [1.25, 4.08]	0.007*
4 [0.25, 31.17]	10/60	23.4		3.45 [1.54, 7.73]	0.003*	2.56 [1.10, 5.95]	0.029*
Relative change as a continuous variable^c^				1.04 [1.02, 1.06]	<0.001*	1.03^c^ [1.01, 1.05]	0.001*

^a^ Minimum and maximum level for relative level given for each category

^**b**^ adjusted for log-transformed baseline levels of NT-proBNP, age, sex, BMI, blood pressure, LV function, current smoking, total cholesterol, HDL-cholesterol, GFRstd, and use of statins

^**c**^ A 10% increment of ln NT proBNP, equivalent to 10% increment of absolute NT-proBNP, is associated with a HR 1.35 [1.12, 1.63] with the occurrence of an adverse CVE.

Addition of the 12-month relative change of ln NT-proBNP in categories to our **basic baseline prognostic model** increased the C statistic from 0.69 [95% CI 0.64, 0.74] to 0.71 [95% CI 0.66, 0.76]. In terms of reclassification, this addition did not provide a net upwards reclassification among the cases (-0.009). However a net downwards reclassification among the non-cases equal to 0.086 could be detected, for a NRI of 0.077 [95% CI −0.015, 0.170] (**Table 3**).

## Discussion

The present study among patients with stable CHD showed that besides NT-proBNP measured at baseline, NT-proBNP measured one year later not only remains an important predictor of subsequent CVD events but also allows an improvement in the risk stratification independent of LV function and other established CV risk factors. Therefore, changes over 12-months may help to identify patients at special risk for an adverse secondary CVE, who could require closer follow-up evaluations.

Several observational cohorts including more than 12,000 patients have demonstrated that higher levels of NT-proBNP are associated with higher risk for mortality[15]. In 2006 we examined the prognostic value of NT-proBNP with respect to subsequent events within 4.5 years of follow-up, demonstrating the superior predictive utility of NT-proBNP when compared to CRP, and creatinine clearance[21]. In 987 patients with stable CHD, NT-proBNP was also able to predict subsequent CVD events and death independent of traditional CV risk factors[22]. NT-proBNP was the only biomarker among a panel of nine inflammatory biomarkers able to predict adverse CVE (both fatal and non-fatal) independent of traditional risk factors in the HOPE trial[23]. Among 756 older men with CVD but without overt HF participating in the British Regional Heart Study, high levels of NT-proBNP were associated with major CVE, CV mortality, major CHD events, and fatal CHD after adjustment for traditional CV risk factors, with a statistically significant improvement of NRI[24], while baseline NT-proBNP levels showed a prognostic value with respect to long-term mortality after adjustment for conventional CV risk factors and the degree of LV function among 1034 patients with stable CHD[25].

High levels of NT-proBNP in asymptomatic patients could reflect subclinical levels of ventricular dysfunction or inducible ischemia[26,27,28], as well as the presence of vascular dysfunction or atherosclerosis progression[15]. Elevated levels of NT-proBNP have been independently associated with the development of HF among subjects with normal LV function at baseline[27]. Among cardiac outpatients normal NT-proBNP levels have been associated with an excellent prognosis irrespective of any echocardiographic findings, and in addition, NT-proBNP had been able to discriminate between those with and without a CVE in those subjects with abnormal echocardiographic findings[29].

In our cohort we noted markedly elevated NT-proBNP levels at the end of the cardiac rehabilitation program, most likely corresponding to the second peak registered usually weeks after the initial event, and which may reflect the reversible increase in regional wall stress due to impaired LV function[15,21]. Our analysis did not show any association between the measured levels of NT-proBNP and the time since acute event. The high baseline levels of NT-proBNP tended to normalize at one-year follow-up, and a 12-month 10% increment of NT-proBNP is associated with a 35% higher risk for an adverse CVE in patients with CHD.

Recent developments in the evaluation of the prognostic value of a biomarker show the introduction of the Net Benefit Analysis, which considers the utility of the addition of a novel biomarker on an absolute scale[30]. When applied to our modest sample size the model containing the baseline levels of NT-proBNP showed the highest increment of the fraction of true positives identified in this population, without a change in false positive (7 per 1000, compared to 2 per 1000 in the model containing 12-months change of ln NT-proBNP as a continuous variable) (**S3 Table**). Further research is needed in order to evaluate the accuracy of this proposed method.

Our data based on a modest sample size suggest that among patients with a history of CHD, an additional NT-proBNP measurement during 12-months follow-up could offer a better prognostic value by helping to identify subjects at high-risk, who could benefit from closer follow-ups including the implementation of possible novel CVD protective modalities[31,32]. Further research evaluating the effect of longitudinal measurements of NT-proBNP in stable cardiac patients is needed.

### Strengths and Limitations

Strengths in our study include the long-term and high completeness of ten years follow-up. Some limitations warrant mentioning. Our results were based on one occasion NT-proBNP measurements for both time points, so that day-to-day variability could not be assessed. Our analyses were adjusted using baseline covariates levels, however the performed analyses reflect the clinical practice, where depending on the location of the follow-up examinations not always the updated information on all covariates of interest may be available. As this was an observational study, possible presence of residual confounding should be considered. Our study consisted mostly of male, Caucasian patients (84.5%). Case numbers were insufficient to perform separate analyses by sex and other ethnic/racial groups, as well as for each of the single adverse events: fatal MI, fatal stroke, non-fatal MI, and non-fatal stroke. Not all patients participate in cardiac rehabilitation, so that we could be missing the most severe cases in our cohort, affecting the generalizability of the results. Nevertheless this should not affect the internal validity of our data, suggesting also that the true prognostic value of NT-proBNP may indeed be stronger.

### Conclusions

One-year follow-up measurements of NT-proBNP after an acute CVE may provide a numerically better discrimination and classification of patients at risk for an adverse CVE compared to baseline values obtained shortly after the acute event. Twelve-month longitudinal changes of NT-proBNP in patients with CHD not only remain strongly associated with subsequent adverse CVE but also improve risk classification, albeit only modestly, after its inclusion in a prognostic model containing well established risk factors.

## Supporting Information

S1 Table Sociodemographic, clinical and laboratory characteristics of participants. (DOC)

S2 Table NT-proBNP distribution (median) according to various sociodemographic characteristics, cardiovascular risk factors, and discharge medication (end of rehab) at baseline and at one-year follow-up (N = 798). (DOC)

S3 Table Risk tables for reclassified persons based on a >20% 10-year CHD risk threshold to define low and high risk for a subsequent cardiovascular event. (DOC)
