# Supplementary material for: Serial Measurements of N-Terminal Pro-Brain Natriuretic Peptide in Patients with Coronary Heart Disease
Source: PLoS One. 2015 Jan 28;10(1):e0117143. doi: 10.1371/journal.pone.0117143 (PMC4309398; doi:10.1371/journal.pone.0117143)
Supplement: S1 Table — (DOC) [file pone.0117143.s001.doc]

| **Table S1.** Sociodemographic, clinical and laboratory characteristics of participants | | |
| --- | --- | --- |
|  | **Study Sample (n=798)** | **With follow-up < one year (n=210)** |
| **Age** (year) mean ± std | 59 ± 8 | 58 ± 9 |
| **Male** (%) | 676 (84.7) | 167 (79.5) |
| **Body Mass Index** (kg/m^2^) mean ± std | 27.0 ± 3.5 | 27.3 ± 3.8 |
| **Body Mass Index** n (%) |  |  |
| Normal weight | 221 (27.7) | 56 (26.7) |
| Overweight | 443 (55.5) | 104 (49.5) |
| Obese | 134 (17) | 50 (23.8) |
| **Smoking** n (%) |  |  |
| Never smoker | 262 (32.8) | 47 (22.4) |
| Ex-smoker | 505 (63.3) | 144 (68.6) |
| Current smoker | 31 (3.9) | 19 (9.1) |
| **History of Myocardial Infarction** n (%) | 405 (50.8) | 109 (51.9) |
| **Coronary Artery Bypass Graft** n (%) | 395 (49.5) | 85 (40.5) |
| **History of Diabetes** n (%) | 129 (16.2) | 51 (24.3) |
| **Left Ventricular Function** n (%) |  |  |
| Normal | 434 (54.4) | 86 (45.03) |
| Mild depression | 196 (24.6) | 53 (27.8) |
| Moderate depression | 122 (15.3) | 40 (20.9) |
| Severe depression | 46 (5.8) | 12 (6.3) |
| **Systolic Blood Pressure** (mmHg) mean ± std | 120 ± 15 | 121 ± 15 |
| **Diastolic Blood Pressure** (mmHg) mean ± std | 73 ± 9 | 74 ± 9 |
| **Blood Pressure^a^** n (%) | | |
| 0 points | 522 (65.4) | 130 (61.9) |
| 1 point | 133 (16.7) | 30 (14.3) |
| 2 points | 129 (16.2) | 44 (20.9) |
| 3 points | 14 (1.8) | 6 (2.9) |
| **Time to blood withdrawal** (median, q1-q3) | 43 (36, 52) | 45 (37, 54) |
| **Total Cholesterol** (mg/dl) mean ± std | 168 ± 32 | 180 ± 40 |
| **HDL-cholesterol** (mg/dl) mean ± std | 40 ± 11 | 41 ± 13 |
| **NT-proBNP** (pg/mL) (median, (interquartile range Q1, Q3)) |  |  |
| Female | 690.2 (357.7, 1644) | 836.9 (368.2, 1546) |
| Male | 542.5 (277.6, 1043.5) | 592.3 (247.8, 1274) |
| ^a^according to FRS chart for 10 years risk for CHD | | |
