# Supplementary material for: Serial Measurements of N-Terminal Pro-Brain Natriuretic Peptide in Patients with Coronary Heart Disease
Source: PLoS One. 2015 Jan 28;10(1):e0117143. doi: 10.1371/journal.pone.0117143 (PMC4309398; doi:10.1371/journal.pone.0117143)
Supplement: S2 Table — (DOC) [file pone.0117143.s002.doc]

| **Table S2.** NT-proBNP distribution (median) according to various sociodemographic characteristics, cardiovascular risk factors, and discharge medication (end of rehab) at baseline and at one-year follow-up (N=798) | | | | | |
| --- | --- | --- | --- | --- | --- |
|  |  | **Baseline** | | **One-year follow-up** | |
|  | n | NT-proBNP (pg/mL) Median | *P*-value | NT-proBNP (pg/mL) Median | *P*-value |
| **Sex** | | | | | |
| Male | 676 | 542.5 | 0.002^a^ | 179.5 | 0.002 ^a^ |
| Female | 122 | 690.2 |  | 270 |  |
| **Age (years)** | | | | | |
| 30-39 | 15 | 185 | <0.001^b^ | 53.2 | <0.001 ^b^ |
| 40-49 | 97 | 361.9 |  | 102 |  |
| 50-59 | 229 | 433.4 |  | 136 |  |
| 60-70 | 457 | 687.9 |  | 264 |  |
| **Time to blood withdrawal (days)** | | | | | |
| Q1 [30 – 35] | 201 | 602 | 0.944 ^b^ |  |  |
| Q2 [38 – 42] | 205 | 543.7 |  |  |  |
| Q3 [46 – 50] | 196 | 556.45 |  |  |  |
| Q4 [56 - 70] | 196 | 562.9 |  |  |  |
| **Body Mass Index (kg/m^2^)** | | | | | |
| < 25 | 221 | 648.3 | <0.001 ^b^ | 256 | <0.001 ^b^ |
| 25 – 30 | 443 | 543.7 |  | 172 |  |
| > 30 | 134 | 475.6 |  | 177 |  |
| **GFR std** | | | | | |
| Q1 [-4.69, -0.53] | 199 | 885.4 | <0.001 ^b^ | 366 | <0.001 ^b^ |
| Q2 [-0.52, 0.17] | 200 | 606.8 |  | 223 |  |
| Q3 [0.17, 0.70] | 200 | 547.2 |  | 157 |  |
| Q4 [0.70 – 2.39] | 199 | 361.9 |  | 116 |  |
| **Left Ventricular Function** | | | | | |
| Normal | 434 | 410.2 | <0.001 ^b^ | 144 | <0.001 ^b^ |
| Mild depression | 196 | 579.9 |  | 184.5 |  |
| Moderate depression | 122 | 1095 |  | 346.5 |  |
| Severe depression | 46 | 2010 |  | 753 |  |
| **HDL (mg/dL)** | | | | | |
| Q1 [5 – 32] | 202 | 604.3 | 0.576 ^b^ | 176.5 | 0.807 ^b^ |
| Q2 [33 – 39] | 211 | 589.8 |  | 184 |  |
| Q3 [40 – 46] | 192 | 543.1 |  | 220.5 |  |
| Q4 [47 – 118] | 193 | 501.9 |  | 184 |  |
| **Total Cholesterol (mg/dL)** | | | | | |
| Q1 [90 – 145] | 201 | 587.3 | 0.672 ^b^ | 186 | 0.672 ^b^ |
| Q2 [146 – 164] | 196 | 603.8 |  | 198 |  |
| Q3 [165 – 185] | 198 | 517.1 |  | 165.5 |  |
| Q4 [186 – 334] | 203 | 559.5 |  | 215 |  |
| **History of Diabetes** |  |  |  |  |  |
| Yes | 129 | 661 | 0.071 ^a^ | 255 | 0.027 ^a^ |
| No | 669 | 557.3 |  | 178 |  |
| **Blood Pressure^c^** |  |  |  |  |  |
| 0 | 522 | 544.9 | 0.135 ^b^ | 168.5 | 0.006 ^b^ |
| 1 | 133 | 553.5 |  | 191 |  |
| 2 | 129 | 645.2 |  | 234 |  |
| 3 | 14 | 814.2 |  | 270.5 |  |

| **Table S2 (cont.).** NT-proBNP distribution (median) according to various sociodemographic characteristics, cardiovascular risk factors, and discharge medication (end of rehab) at baseline and at one-year follow-up (N=798) | | | | | |
| --- | --- | --- | --- | --- | --- |
|  |  | **Baseline** | | **One-year follow-up** | |
|  | n | NT-proBNP (pg/mL) Median | *P*-value | NT-proBNP (pg/mL) Median | *P*-value |
| **Current smoker** | 31 | 718 | 0.274 ^b^ | 171 | 0.347 ^b^ |
| **Use of Statins** | | | | | |
| Yes | 614 | 546.5 | 0.017 ^a^ | 180.5 | 0.005 ^a^ |
| No | 184 | 628.2 |  | 249 |  |

^a^ Wilcoxon rank sum test

^b^ Kruskal-Wallis test
^c^ According to FRS chart for estimation of 10 years risk for CHD
