# Supplementary material for: Serial Measurements of N-Terminal Pro-Brain Natriuretic Peptide in Patients with Coronary Heart Disease
Source: PLoS One. 2015 Jan 28;10(1):e0117143. doi: 10.1371/journal.pone.0117143 (PMC4309398; doi:10.1371/journal.pone.0117143)
Supplement: S3 Table — (DOC) [file pone.0117143.s003.doc]

**Table S3**

| Risk table for reclassified persons with the addition of baseline levels of NT-proBNP to the baseline prognostic model, based on a >20% 10-year CHD risk threshold to define low and high risk. | | | |
| --- | --- | --- | --- |
| **Reclassification** | **Events** | **Nonevents** | **N** |
| Low 🡪 Low | 25 | 383 | 408 |
| High 🡪 Low | 5 | 38 | 43 |
| Low 🡪 High | 11 | 36 | 47 |
| High 🡪 High | 73 | 227 | 300 |
| Total | 114 | 684 | 798 |

NRI events= (11-5)/114=5.26%

NRI nonevents=(38-36)/684=0.29%

NRI=5.26%+0.29%= 0.0555

NB=(6-0.25x2)/798=0.00689

RU=(6-0.25x2)/114=0.0482

| Risk table for reclassified persons with the addition of one-year levels of NT-proBNP to the baseline prognostic model, based on a >20% 10-year CHD risk threshold to define low and high risk. | | | |
| --- | --- | --- | --- |
| **Reclassification** | **Events** | **Nonevents** | **N** |
| Low 🡪 Low | 25 | 369 | 394 |
| High 🡪 Low | 7 | 66 | 73 |
| Low 🡪 High | 11 | 50 | 61 |
| High 🡪 High | 71 | 199 | 270 |
| Total | 114 | 684 | 798 |

NRI events= (11-7)/114=3.5%

NRI nonevents=(66-50)/684=2.3%

NRI=3.5%+2.3%= 0.058

NB=(4-0.25x16)/798=0

RU=(4-0.25x16)/114=0

| Risk table for reclassified persons with the addition of change of ln NT-proBNP and baseline levels of ln NT-proBNP to the baseline prognostic model, based on a >20% 10-year CHD risk threshold to define low and high risk. | | | |
| --- | --- | --- | --- |
| **Reclassification** | **Events** | **Nonevents** | **N** |
| Low 🡪 Low | 25 | 369 | 394 |
| High 🡪 Low | 7 | 61 | 68 |
| Low 🡪 High | 11 | 50 | 61 |
| High 🡪 High | 71 | 204 | 275 |
| Total | 114 | 684 | 798 |

NRI events= (11-7)/114=3.5%

NRI nonevents=(61-50)/684=1.61%

NRI=3.5%+1.61%= 0.051

NB=(4-0.25x11)/798=0.0016

RU=(4-0.25x11)/114=0.01096
